# Albumin, copper, manganese and cobalt levels in children suffering from sickle cell anemia at Kasumbalesa, in Democratic Republic of Congo

**DOI:** 10.1186/s12878-018-0118-z

**Published:** 2018-09-06

**Authors:** Olivier Mukuku, Joseph K. Sungu, Augustin Mulangu Mutombo, Paul Makan Mawaw, Michel Ntetani Aloni, Stanislas Okitotsho Wembonyama, Oscar Numbi Luboya

**Affiliations:** 1 Department of Research, High Institute of Techniques Medicales, Lubumbashi, Democratic Republic of Congo; 2 Department of Pediatrics, University Hospital of Lubumbashi University of Lubumbashi, Lubumbashi, Democratic Republic of Congo; 3 School of Public Health, University of Lubumbashi, Lubumbashi, Democratic Republic of Congo; 4 Division of Hemato-oncology and Nephrology, Department of Paediatrics, University Hospital of Kinshasa, School of Medicine, University of Kinshasa,, Kinshasa, Democratic Republic of Congo

**Keywords:** Sickle cell Anemia, Children, Trace elements, Africa

## Abstract

**Background:**

Sickle Cell Anemia (SCA) is characterized by high levels of oxidative stress markers and low levels of antioxidant capacity. Antioxidant defence mechanisms against the harmful effects of ROS requires cellular and extracellular enzymes. These enzymes requires micronutrient for complete activity. Information on micronutrients such as manganese, cobalt and copper in SCA population was poorly documented in the literature.

**Methods:**

Plasma copper, manganese, cobalt and albumin concentrations determined by atomic absorption spectrophotometry were compared between two groups of children: 76 with SCA (Hb-SS) and 76 without SCA (controls). This study was conducted in the Muhona Hospital of Kasumbalesa, which is situated in a rural and low in resources.

**Results:**

The mean age was 10.0 years (SD = 5.4) in SCA children and 9.2 years (SD = 4.7) in the control group. The levels of cobalt, manganese, copper and albumin were not different between the two groups (*p* > 0.05).

**Conclusion:**

In our study, albumin, manganese, cobalt and copper values did not differ between SCA children in steady state and Hb-AA children. The lack of differences in plasma elemental concentrations between the two groups in context of increased demands in the SCA group, may represent adequate compensatory intake or elemental dyshomeostasis in the SCA group.

## Background

Sickle cell anaemia (SCA) remains the most common genetic diseases and major problem in public health in the world. The incidence is estimated to range from 30,000 to 40,000 neonates per year in a recent report [1].

The disease is characterized by chronic hemolysis, chronic inflammation, immune deficiency, a heterogeneous clinical phenotype and organ damage [2–7]. Pathogenic mechanism in sickle cell disease is mainly due to chronic inflammation with oxidative stress. This situation leads to high levels of oxidative stress markers and low levels of antioxidant capacity in SCA patients. Antioxidant defence mechanisms against the harmful effects of reactive oxygen species (ROS) requires cellular and extracellular enzymes such as peroxidase, glutathione reductase, catalase and superoxide dismutase (SOD). These enzymes require micronutrients for complete activity.

SCA is associated with increased risks of multiple micronutrient deficiencies but no significant differences found in the levels of copper and albumin in SCA adults compared to Hb-AA adults [8]. These nutriments have a major role in the protection of the red cell membrane against stress free radical mediated by oxidation in SCA [9–11]. Children suffering from SCA have significantly lower serum levels of zinc, magnesium and selenium [12]. SOD is a copper and zinc-containing enzyme that converts superoxide radicals to hydrogen peroxides. Copper is essential for this enzyme’s catalytic activity and antioxidant functions; it plays an important role in the functions of cytochrome c oxidase [13]. A recent study shows a correlation between the oxidant/antioxidant imbalance and alteration in the serum copper level in patients suffering from SCA [14]. A copper excess may contribute to free radical production and oxidative damage [2].

This study is part of a project to evaluate micronutrients in sickle-cell anemia in the African environment. A first study showed that zinc, selenium and magnesium values were significantly lower in SCA children compared to children with normal hemoglobin (Hb-AA) [12]. The objective of this second study was to determine the cobalt, copper, manganese and albumin serum levels among SCA children in steady state. The findings could be a starting point for future research in the understanding of the disease in a context of tropical, malnutrition and in highly resource-scarce settings environment such as the Democratic Republic of Congo (DRC).

## Methods

This study was conducted from January 2014 to June 2014 in the Muhona Hospital of Kasumbalesa, which is situated in the southeastern part of the DRC. This hospital receives all SCA children from the health area of Kasumbalesa which is a rural and low in resources.

We consecutively recruited SCA children (Hb-SS) between the age of 2 years and 15 years after written informed consent provided by their legal guardians. All SCA children were in steady state, free of pain for at one month and had not been hospitalized or transfused for at least 100 days before the study [6]. We excluded subjects (i) under iron therapy (ii) under chronic transfusion program. For each case, one control patient (Hb-AA) matched for age, sex and place of residence were recruited into the study, and 76 SCA children were compared to 76 Hb-AA children.

### Data collection procedure and blood analysis

Five ml of venous blood sample was drawn from each study participant into an EDTA tube, used to determine laboratory parameters.

Five ml of venous blood sample was drawn from each study participant into an EDTA tube, used to determine hemoglobin electrophoresis. Sickle cell screening was performed using isoelectric focusing method.

Cobalt, manganese and copper levels were estimated in blood samples using a Perkin Elmer Model 2380 Atomic absorption Spectrophotometer (Norwak, Connecticut, USA). Albumin was measured in blood samples by the bromocresol green dye binding method. Trace elements were performed at Mineralogy Laboratory of the Société de Développement Industriel et Minier du Congo (SODIMICO) at Kasumbalesa, in DRC.

### Ethics statement

The approval to conduct the study and authorizations were obtained from the Medical Ethic Committee of the University of Lubumbashi (UNILU/CEM/048/2015). Data was used with high confidentiality and no names were recorded.

### Data management and analysis

Results were analyzed using the Epi Info 7.1 (CDC Atlanta, USA) and they were exported on STATA 12 for further analysis. Data are represented as means ± standard deviation (SD) when the distribution was normal and median with range when the distribution was not normal. The analysis of Student’s t-test was used for comparisons of means. Chi square test was also used to compare the difference between groups regarding age groups and gender. Statistical significance level was set at *p* < 0.05.

## Results

A total of 152 children, 76 SCA children and 76 no-SCA children were recruited into the study over the 6 months. The mean age of the Hb-SS group was 10.0 (SD = 5.4) years while that of the Hb-AA group was 9.2 (SD = 4.7) years. The sex-ratio male to female in the SCA group and control group was respectively 1:1.8 and 1:1.1 (Table 1).

**Table 1:** Demographics characteristics of the two study groups

Variable	Hb-SS group (*n* = 76)	Hb-AA group (n = 76)	*p*
Age			
< 5 years	15 (19.7%)	17 (22.4%)	0.7195
5–9 years	21 (27.6%)	24 (31.6%)	
≥ 10 years	40 (52.6%)	35 (46.0%)	
Mean ± SD	10.0 ± 5.4	9.2 ± 4.7	0.3324
Sex			
Female	49 (64.5%)	40 (52.6%)	0.1877
Male	27 (35.5%)	36 (47.4%)	

**Fig. 1:**
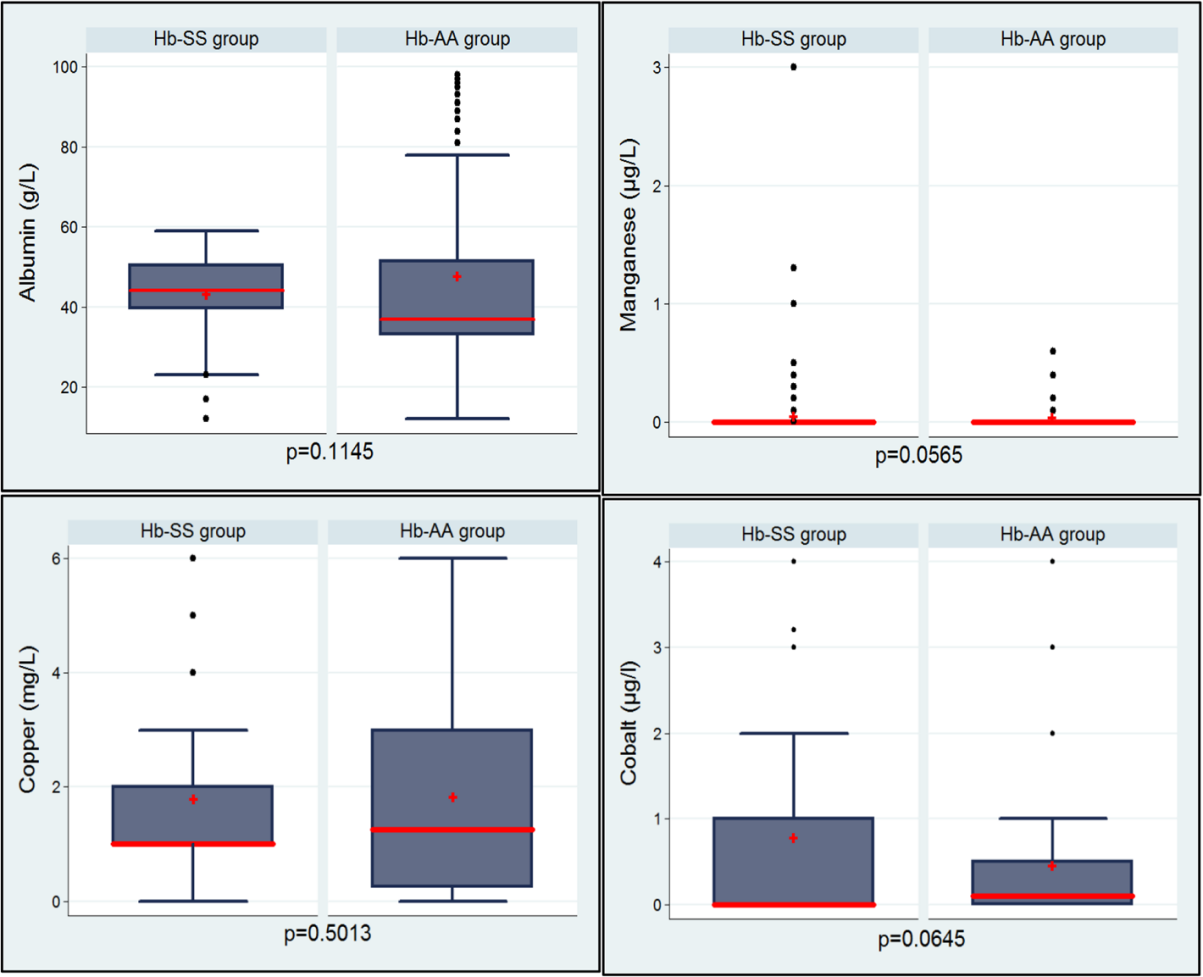
Albumin, manganese, copper and cobalt levels according hemoglobin status. Red line: median; Red cross: means; Line at the furthest lower of the box: first quartile (25%); Line at the furthest upper of the box: third quartile (75%); Dot: outlier; Line at the furthest lower: lowest value (minimum) excluding outliers; Line at the furthest upper: highest value (maximum) excluding outliers. Student’s t-test was used to compare the two groups.

The Fig. 1 shows that the mean albumin level in Hb-SS group tended to be lower than in Hb-AA group. However, there was no statistically difference between the two groups (43.71 ± 1.10 g/L vs 48.46 ± 2.78 mg/L; *p* = 0.50).

In the Hb-SS group, the mean value of manganese was similar to that of the Hb-AA group (0.12 ± 0.04 μg/L vs 0.03 ± 0.01 μg/L; *p* = 0.057) as shown in the Fig. 1.

In the Hb-SS group, the mean cobalt level tended to be higher than in the Hb-AA group (0.81 ± 0.13 μg/L vs 0.48 ± 0.10 μg/L; *p* = 0.065). However, there was no statistical difference between the two groups (Fig. 1).

The Fig. 1 shows that the mean copper level did not vary significantly between the two groups (1.72 ± 0.15 mg/L vs 1.89 ± 0.20 mg/L; *p* = 0.50).

## Discussion

Our study is the first to look at albumin, manganese, cobalt and copper values in SCA children living in the Central Africa. Albumin exerts its anti-oxidant effect in the body by binding copper ions and heme tightly and iron ions weakly. It thus plays a role in preventing copper and iron from participating in lipid peroxidation. In our series, there was no significant difference in the levels of albumin in the SCA patients compared to controls. Our findings are in consonance with previous reports from Nigeria and Sudan [4].

Manganese is a trace element that is required as a cofactor for many antioxidant enzymes such as glutathione peroxidase and superoxide dismutase [5–7]. Thus, this trace element plays a key role in oxidative damage without deleterious side effects of Fenton chemistry. In the SCA group, the mean values of manganese were similar to those of the control group. In his study, Kehinde et al. in Nigeria reported similar findings [15]. By contrast, in their Nigerian series, Digban et al. found that manganese level was significantly decreased in sickle cell patients when compared with apparent controls [16]. On the other hand, Kehinde et al. reported that manganese values were higher in the SCA patients in crisis [15]. We postulate that the metabolism of manganese and are not influenced by the presence of sickle cell anemia in steady state.

In the SCA group, the mean values of cobalt were similar to those of the control group. There is still a lack of information on cobalt in SCA children in the medical literature. Cobalt is an essential element, but at high concentrations, possesses the ability to produce reactive radicals such as superoxide anion radical and nitric oxide in biological systems. This oxidative stress contributes to cell toxicity and death [9, 10]. Cobalt is integral part of vitamin B_12_, its alone function known in human physiology. In the SCA group, the median values of cobalt were similar between sickle cell patients and children with Hb-AA. By contrast, in their Nigerian series, Digban et al. found that cobalt level was significantly decreased in sickle cell patients when compared with apparently controls [16]. There is still a lack of information on cobalt in SCA children in the medical literature. We postulate that the metabolism of cobalt are not influenced by the presence of sickle cell anemia or may be due to normal dietary intake of vitamin, in our series. In addition, we speculate that environment and genetic factors may explain this difference between these two studies.

In this study, the mean copper level did not vary significantly between the two groups, which is similar to the findings reported by Alayash et al. in Saudi Arabia, and by Kehinde et al. in Nigeria [11, 15]. However, our results are different with the previous worldwide reports in which they found a significantly higher serum copper in sickle cell patients compared with controls [2, 14, 17–19]. We therefore speculate that environment and genetic factors may explain this difference between these studies. In addition, rates of child malnutrition remain very high in the DRC in general and particularly in the health zone of Kasumbalesa, which is a rural and low in resources [20].

## Conclusion

The first literature on the subject of albumin, manganese, cobalt and copper values in SCA is briefly reported in Africa. In our study, albumin and these trace elements did not differ between SCA children in a steady state and Hb-AA children. The lack of differences in plasma elemental concentrations between the two groups in context of increased demands in the SCA group, may represent adequate compensatory intake or elemental dyshomeostasis in the SCA group. Further investigations will focus on data on these trace elements in SCA children in crisis compared to those in steady state.

## Abbreviations

DRC: Democratic Republic of Congo
Hb-AA: Hemoglobin AA
Hb-SS: Hemoglobin SS
ROS: Reactive oxygen species
SCA: Sickle Cell Anemia
SD: Standard deviation
SOD: Superoxide dismutase
